# Are fish immunocompetent enough to face climate change?

**DOI:** 10.1098/rsbl.2023.0346

**Published:** 2024-02-21

**Authors:** Andrea Franke, Anne Beemelmanns, Joanna J. Miest

**Affiliations:** ^1^ Helmholtz Institute for Functional Marine Biodiversity at the University of Oldenburg (HIFMB), 26129 Oldenburg, Germany; ^2^ Alfred-Wegener-Institute, Helmholtz-Centre for Polar and Marine Research (AWI), 27570 Bremerhaven, Germany; ^3^ Institut de Biologie Intégrative et des Systèmes (IBIS), Université Laval, G1V0A6 Québec, Canada; ^4^ School of Psychology and Life Sciences, Canterbury, Kent CT1 1QU, UK; ^5^ School of Science, University of Greenwich, Chatham Maritime, Kent ME4 4TB, UK

**Keywords:** immune response, multiple environmental stressors, temperature, hypoxia, disease, epigenetics

## Abstract

Ongoing climate change has already been associated with increased disease outbreaks in wild and farmed fish. Here, we evaluate the current knowledge of climate change-related ecoimmunology in teleosts with a focus on temperature, hypoxia, salinity and acidification before exploring interactive effects of multiple stressors. Our literature review reveals that acute and chronic changes in temperature and dissolved oxygen can compromise fish immunity which can lead to increased disease susceptibility. Moreover, temperature and hypoxia have already been shown to enhance the infectivity of certain pathogens/parasites and to accelerate disease progression. Too few studies exist that have focussed on acidification, but direct immune effects seem to be limited while salinity studies have led to contrasting results. Likewise, multi-stressor experiments essential for unravelling the interactions of simultaneously changing environmental factors are still scarce. This ultimately impedes our ability to estimate to what extent climate change will hamper fish immunity. Our review about epigenetic regulation mechanisms highlights the acclimation potential of the fish immune response to changing environments. However, due to the limited number of epigenetic studies, overarching conclusions cannot be drawn. Finally, we provide an outlook on how to better estimate the effects of realistic climate change scenarios in future immune studies in fish.

## 1. Changing climate, changing fish immunity?

Teleost fish are often keystone species and at the same time essential for food security, livelihoods and cultural practices all over the world. Fish play a central role in food webs and are of high commercial importance in artisanal and/or industrial fisheries and aquaculture. According to the UN Food and Agriculture Organization (FAO), about 600 million livelihoods depend, at least partially, on fisheries and aquaculture, and in many countries, fish is an essential source of protein [1].

Over the twenty-first century, the ocean is projected to transition to unprecedented conditions with further increasing temperatures, acidification and likely oxygen decline due to ongoing anthropogenic induced climate change [2]. Climate change-related effects, especially the warming of aquatic environments, have been associated with an increase in disease outbreaks in wild fish and aquaculture settings (reviewed in [3,4]). However, for most fish species, little is known about how alterations in temperatures, oxygen levels, pH and salinity affect their immune system and hence disease resistance. Current studies on the effects of warming, hypoxia and acidification mainly focus on survival, growth and physiological parameters of fish and very rarely consider more than one environmental stressor. These studies show that the tolerance range to an environmental factor is species or even population specific. Within this range the animal can experience optimal, pejus or critical conditions. In optimal conditions the animal is not stressed and can display maximum performance and fitness. At pejus, conditions are suboptimal and are starting to be stressful for the animal. With increasingly suboptimal conditions, the performance of the animal becomes more and more impaired. At the critical point in the tolerance range survival is time limited and prolonged exposure will result in the death of the animal (see Shelford's law of tolerance and [5]). Short-term acclimation and long-term adaptation can lead to a shift in these tolerance ranges, e.g. leading to a reduced stress response at pejus temperatures or a shift in optimal temperature range [6].

**Figure 1.**
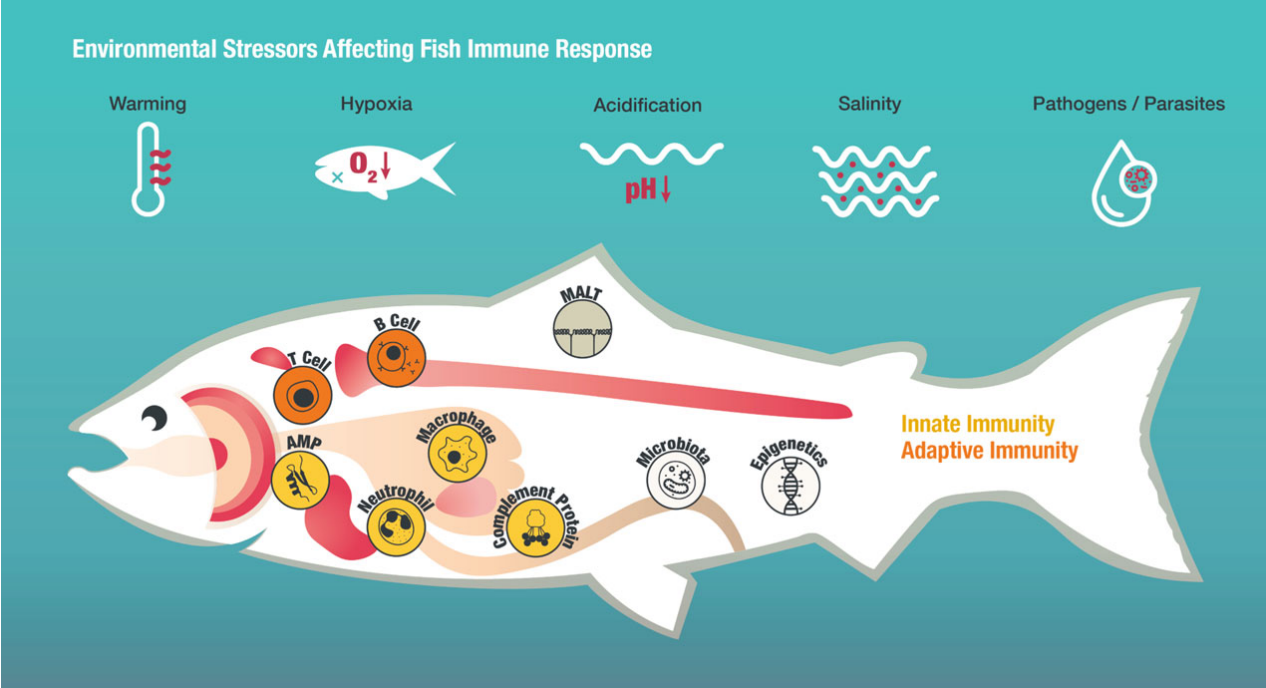
The immune system of fish encompasses innate and adaptive immunity. Important immune organs are thymus, head kidney, spleen, and liver. The mucosa-associated lymphoid tissue (MALT) found in the skin, gut, and gills is another essential part of the fish immune system and is associated with microbial communities that play an important part in shaping the immune response and providing protection against pathogens. Innate immunity is represented here by antimicrobial peptides (AMPs), complement proteins, neutrophils, and macrophages. T and B cells, produced in the thymus and head kidney, form the adaptive immune system. Environmental stressors such as warming, hypoxia, acidification, changes in salinity and pathogens have been shown to affect the immune response in fish on various levels. Epigenetic components are involved in regulating the immunocompetence of fish.

An overview of the fish immune system and the potential environmental stressors influencing it are given in figure 1. The immune system of fish consists of linked innate and adaptive immunity, both encompassing cellular and humoral components [7]. However, early life stages rely solely on innate immunity and maternally transferred immune molecules during their first weeks of development until adaptive immune cells have developed [8,9]. Mucosa-associated lymphoid tissues (MALTs) are distributed in the epithelial mucosa of the skin, the gills, the digestive tract and the nasopharynx. They comprise an array of innate and adaptive immune cells and molecules acting in concert to protect the host against pathogenic invasion [10]. Besides MALTs, central lymphoid organs such as thymus, head kidney, spleen and liver play a crucial role in the fish immune system [11]. Preformed soluble proteins, including antimicrobial enzymes (e.g. lysozyme), antimicrobial peptides (AMPs) and complement proteins, digest or lyse bacteria directly [12]. Innate immune cells include phagocytic cells (e.g. monocytes, macrophages), non-specific cytotoxic cells (NCC) and granulocytes (e.g. neutrophils) (for review see [13]). These cells recognize conserved microbe- or pathogen-associated molecular patterns (MAMPs or PAMPs) by means of pattern-recognition receptors (PRRs) such as toll-like receptors (TLRs) [14]. Activation of the cell induces downstream signalling pathways resulting, among others, in the production of cytokines such as interleukins, which regulate a variety of cell functions [15]. This first line of defence is quick and unspecific. By contrast, the adaptive immune system reacts slowly (depending on the environmental temperature within days or weeks) but is highly specific and facilitates immunological memory [16]. Lymphocytes (T and B cells) recognize a diverse variety of different antigens from pathogens, with the consequence that infected cells and extracellular pathogens are eliminated [17]. B cells produce antigen-specific recognition proteins in the form of immunoglobulins, which are either membrane-bound or secreted as soluble antibodies [11,18]. T cells can detect the presence of intracellular pathogens and eliminate infected cells [19]. Molecular fractions of antigens (i.e. epitopes) are presented on the surface of cells by two different classes of major histocompatibility complex (MHC) molecules: MHC I and MHC II. MHC I is expressed on the surface of all nucleated cells and presents epitopes of internal pathogens to killer T cells, which will then kill the infected host cell. MHC II is expressed by antigen-presenting cells such as macrophages, dendritic cells and B cells and presents epitopes to T helper cells. Upon antigen recognition, T helper cells activate B cells to produce antibodies and macrophages to phagocytose foreign particles [20,21]. An exception is the loss of MHC II receptors in cod-like fishes (family Gadidae), certain pipefishes (genus *Syngnathus*) and anglerfish (*Lophius piscatorius*) [22–26].

Here we reviewed the literature regarding the impact of climate change related environmental factors on the immunity of marine and freshwater teleosts with the aim to evaluate if fish will be immunocompetent enough to fight infections in future aquatic environments. Reflecting the current knowledge, the major focus of this review is on temperature and hypoxia effects (including microbiota studies and pathogenic challenges). We also discuss the current knowledge on effects of acidification and salinity before exploring interactive effects of multiple stressors. A special focus of this review is the underlying epigenetic mechanisms of fish immunity to cope with the challenges associated with climate change. Finally, we elucidate which experimental factors should be considered and/or improved to estimate the effects of realistic climate change scenarios in future studies.

## 2. Environmental stressors

Changes in the environment influence the physiology of vertebrate animals to different extents. Physiological stress is caused when the external environment becomes unfavourable to an individual. Urbinati and her colleagues give an excellent review of the teleost stress response and its influence on the immune system [27]. The stress response can be divided into primary (i.e. initial response to an acute stressor), secondary (adaptive response to maintain homeostasis) and tertiary response (exhaustion of biological systems due to strong and/or prolonged stressor). During the primary response an upregulation of the innate immune system is often observed, the secondary response is associated with a modulation of the immune system to restore homeostasis. However, the tertiary response causes immunosuppression due to a reallocation of energy to vital function to ensure survival [27]. As discussed in the present review it is therefore important to consider the strength and duration of each particular stressor as this will influence the immune response.

### (a) Temperature

The global ocean temperature has increased continuously over the last 50 years [2]. According to the Intergovernmental Panel on Climate Change (IPCC), marine heatwaves have ‘very likely’ doubled in frequency and are increasing in intensity since the early 1980s and the rate of ocean warming has ‘likely’ more than doubled since the 1990s [2]. Over the twenty-first century, aquatic environments will continue to warm to an extent that is unprecedented with heatwaves and extreme El Niño and La Niña events becoming more frequent [2]. Consequently, fish will have to cope with continuous long-term warming and additionally more frequent and intense acute heat/cold stress events, affecting their immune system and stress response [28].

Temperature is the major environmental factor influencing the immune response of ectothermic animals, such as fish, with a species-specific optimum for greatest immune activity [29,30]. When comparing the immune response of fish upon exposure to altered temperatures, it needs to be considered that fish differ greatly in the thermal ranges they can endure (habitats range from −2.5°C to 44°C; [31]). Fish species are divided into thermal generalists tolerating highly variable temperatures on daily and seasonal timescales (eurythermal) and thermal specialists with a narrow range of thermal tolerance living at essentially constant temperatures (stenothermal) [32]. Moreover, with an increase in ecoimmunology studies, it has become clear that the effect on fish immune functions varies depending on the duration and magnitude of the temperature change, the resulting stress level (e.g. increased cortisol levels can lead to decreased immunity), the type of pathogen, the fish species/population as well as the life stage and the sex of the fish (sexual immune dimorphism) [33–37]. For an overview on studies examining the effect of temperature on the fish immune response, see electronic supplementary material, table S1 and additionally Scharsack & Franke [38] and Makrinos & Bowden [34].

As ectotherms, many fish species rely on their innate immune system in temperatures below their thermal optimum (e.g. during winter) as T and B cell proliferation and antibody production are inhibited (reviewed in [33]). In temperate climate zones, the immune response is enhanced with warming water temperatures during spring and summer, as long as temperatures are within the species' thermal tolerance range [29,38,39]. Fish can even combat infections behaviourally by increasing immunocompetence when moving into areas with warmer water, which is known as behavioural fever [40]. In the context of climate change with rising mean winter temperatures however it is unclear if the transition from winter to spring will remain immunostimulatory [41].

As described above, climate change increases average water temperatures, yet few experimentally and statistically sound studies have addressed long-term climate change effects on fish immunity and disease susceptibility. Therefore, it is currently difficult to reliably predict disease outcomes in future aquatic environments. If temperatures stay close to the thermal optimum of the fish species, warming can enhance the immune response. For example, elevated antibody levels were found with increasing (but not above optimal) temperatures in catfish (*Ictalurus punctatus*) vaccinated against *Ichthyophthirius multifiliis* 14- and 21-days post injection [42]. However, chronic warming to pejus temperatures has rarely been addressed in respect to immunity but may cause impairments through chronic stress [27]. Atlantic cod (*Gadus morhua*) kept at their upper thermal tolerance level (14°C) over 12 months had a significantly higher mortality rate (compared to control fish kept 7°C) which was mostly associated with parasite (*Loma morhua*) infestation and occasionally with fungal and bacterial infections [43]. However, it is noteworthy that the experiment was outside the scope of future warming scenarios since fish were kept at their upper thermal limit for an entire year and not only throughout a summer season. Hence, knowledge regarding the acclimation and adaptation potential of fish to realistic future changes in temperature is limited (see §5).

Climate change not only leads to elevated mean temperatures but also increases the occurrence of extreme events such as heatwaves and cold snaps, exposing the animals to suboptimal temperatures causing acute physiological stress or even mass mortality events [44]. The already observed higher fluctuations in temperature due to climate change may have complex effects on the immune activity [41,45,46]. Heatwaves may have an overall immunocompromising effect in fish as shown, for example, in three-spined sticklebacks (*Gasterosteus aculeatus,* [47]). When sticklebacks were exposed to 13, 18 (control) and 24°C for up to two weeks, cellular innate immunity (respiratory burst, monocyte proliferation) was downregulated immediately at 24°C [47]. After two weeks, the same parameters were higher at 13°C compared to 18 and 24°C indicating that stickleback immune activity is optimal rather at 13 than at 18°C. A subsequent heat-wave experiment (18°C control, 24 and 28°C for two weeks followed by two-week recovery time) was performed with cold- and warm-adapted stickleback populations. At 28°C, the upper physiological temperature limit of sticklebacks, warm-adapted fish showed only a slightly enhanced innate immune activity directly after the heatwave. By contrast, in cold-adapted fish, a highly increased innate and adaptive immune activity was observed which, according to the authors, was unlikely to result in an upregulated immunocompetence but might rather be a response to cell and tissue damage caused by thermal stress. The recovery period did not lead to a return to baseline levels. Instead, immune activity remained increased, especially in cold-adapted sticklebacks, which may immunocompromise the fish, thereby potentially facilitating the spread of infectious diseases. Overall, this study indicates that warm adaptation can lower immunological vulnerability to heatwaves and provides valuable insights into the immune response of different ecotypes [47]. To understand the species-specific molecular response of five different stenothermal damselfish and cardinalfish species, liver transcriptomes were analysed from fish sampled in the field before (*ca* 28°C), at the beginning (*ca* 29°C), during (*ca* 30°C) and after (*ca* 25°C) the Great Barrier Reef heatwave of 2016 [48]. An activation of complement pathways promoting inflammation during cellular stress as well as changes in the regulation of apoptosis were only found in damselfish, highlighting how species-specific the impact of heatwaves is [48]. Furthermore, in pikeperch (*Sander lucioperca*), short heat stress (30, 32 and 34°C for 2 h, control 23°C) triggered an immune and apoptotic response on the gene expression level [49]. The interconnection of immune and stress response was also demonstrated in juvenile hybrid yellow catfish (*P. fulvidraco* × *P. vachelli*) upon heat stress (35°C for up to 96 h, control 28°C) as complement C3, lysozyme, cortisol and antioxidant enzyme (SOD, CAT) levels were all affected [50]. The results show that heat stress triggered costly metabolic responses depleting the energy reserves of catfish which in turn may lower their immunocompetence. Likewise, short and acute cold stress affects the fish immune response. In pufferfish (*Takifugu obscurus*), a decrease in blood cell count was observed under short cold stress (for 24 h at 21, 17, 13°C, control 25°C) which may lead to an impaired immune response. Moreover, the gene expression of antioxidant enzymes (*sod*, *cat*), *hsp90* and complement *c3* was increased by decreased temperatures and molecular markers for apoptosis were induced [51]. Tilapia (*Oreochromis mossambicus*) exposed to 12°C for up to 48 h (control 24 to 28°C) showed increased lysozyme, phagocytosis, respiratory burst and alternative complement pathway activities in blood plasma suggesting a potential role of humoral innate immune response during cold stress exposure [52]. The discussed studies provide compelling evidence on the interconnection of the stress and immune response upon acute temperature shifts in diverse fish species and highlight the importance of the complement system.

While fish often cannot respond quickly enough to acute temperature events, behavioural thermoregulation has been observed in areas with increasing mean water temperatures with fish moving to colder environments [53–55]. However, the migration to new habitats might bring fish in contact with novel pathogens they have not encountered before, potentially leading to increased disease outbreaks.

Temperature is also one of the environmental key factors impacting the microbiome of fish. The MALT is part of the fish immune system and associated with microbial communities [56]. Microbes play a crucial role in the development and maturation of MALTs, which mediate various host immune functions, especially during early life stages [57]. Hence, the establishment of a functional core microbiota is a key component for excluding pathogenic microbes and maintaining health [10]. That increasing temperatures can negatively affect the microbiome of fish was demonstrated in several studies, e.g. through enhanced microbial growth [44], and/or changes in microbial diversity and community structure in the mucosa [58,59]. In Atlantic salmon (*Salmo salar*), an increase in *Vibrio* numbers and a decrease in lactic acid bacteria (LAB) was observed in the gut in summer [60]. Such changes in the gut microbiota composition could have negative impacts on the fish’ health since LAB can be protective against opportunistic *Vibrio* spp. that can become increasingly virulent at higher temperatures [61,62]. Brown trout (*Salmo trutta*) showed a higher diversity of egg-associated bacteria at elevated temperatures, likely affecting the initial larval microbial colonization during hatching which influences the development of the larval immune system [63]. In farmed fish, the application of immunostimulants may help to steer the gut microbiota towards a healthy community, to exert positive effects on fish immunity, and to increase disease resistance [64–67]. However, the administration of immunostimulatory compounds is not applicable in wild fish.

How host–pathogen interactions will be affected by global warming is another important point to consider. Warming aquatic environments can potentially (i) increase pathogen development and reproduction rates, virulence, pathogenicity, and transmission, (ii) relax overwintering restrictions on pathogen life cycles, and (iii) modulate the host immune function [61,68]. For more details on host–pathogen coevolution in warming environments, see Scharsack & Franke [38]. In three-spined sticklebacks, a model organism for host–parasite interactions in fish, elevated temperatures seem to increase the virulence and expedite the infectivity of the tapeworm *Schistocephalus solidus* while impairing the host′s immune response [38,69,70]. By contrast, in a heatwave experiment (18°C control, 25°C heatwave, duration 3 weeks) with broad-nosed pipefish (*Syngnathus typhle*), temperature had no effect on the infectivity of the trematode *Cryptocotyle lingua* and no interaction between cellular host immune defence, parasite infectivity, and temperature were found [71]. For microparasites it has been shown that global warming increases bacterial replication and virulence and, for example, is driving the emergence of *Vibrio* outbreaks causing vibriosis, a fatal aquatic disease [44,72,73]. This is, not only, but especially problematic in aquaculture settings where high stocking densities already facilitate pathogen proliferation and transmission, and host immunity can be depressed due to crowding stress [74–76]. Thus, disease outbreaks are projected to become more frequent and severe while antimicrobial resistance will likely be further aggravated [77,78]. Furthermore, a pathogen-dependence was shown in juvenile Atlantic cod kept at 10°C (spring temperature) and 16°C (summer temperature) and challenged with bacterial and viral antigens [79]. While the antibacterial response in terms of immune-gene expression in the spleen hardly differed between 10 and 16°C, the antiviral response was affected by the temperature treatment. Accordingly, it is crucial to include a range of relevant pathogens and parasites when trying to understand climate change related immunocompetence in fish.

Another key aspect to consider is that different life stages respond differently to changing temperatures. Early life stages are known to be the most sensitive life cycle stages especially due to their high vulnerability regarding suboptimal temperatures [80] and high susceptibility to infectious diseases as they lack a mature immune system [81]. Fish larvae are solely protected by their innate immune response while their adaptive immune system is still developing [12,82]. Consequently, adverse environmental conditions (such as suboptimal temperatures, hypoxia, etc.) have the severest impact during early development [83]. Since the ontogenetic developmental pattern of the adaptive immune system in fish is species specific, the developmental stage at which fish are immunological mature differs between species. While there is a considerable number of studies on climate change effects on the development of fish larvae (e.g. [84,85]), the impact on larval immune functioning has scarcely been investigated. In European eel (*Anguilla anguilla*), rearing of larvae at temperatures from 16 to 22°C (control 18 to 20°C) influenced the molecular ontogeny of the immune system, which appeared dysregulated at 22°C [86]. In sea bream (*Sparus aurata*), egg and larval rearing at higher than optimal temperatures resulted in a suppression of innate immunity in adults [87]. These studies indicate that the immunocompetence of fish early life stages is potentially lowered at suboptimal temperatures that are likely to occur more and more frequently due to climate change. Additionally, there is an indication of latent effects, possibly caused by epigenetic mechanisms, as described in §4 of this review.

In summary, temperature is the main factor driving the fish immune response. Temperatures outside the thermal optimum can suppress the immune response either directly through the sensitivity of immune factors or indirectly through an increased stress response and/or disadvantageous alterations of the microbiome. Consequently, acute and chronic temperature shifts due to climate change may ultimately lead to immunological disorders and an increased susceptibility to infections in fish. It can be expected that extreme temperature events will have greater effects on stenothermal than on eurythermal fish due to their narrow temperature range. Generally, the impact of changing temperatures will depend on the fish species, the ecotype, the life cycle stage, and the infecting pathogen/parasite. Additionally, many fish species have long generation times and/or are distributed at their upper thermal tolerance limits leading to low potential for evolutionary rescue [88,89] (see §5). Altogether, the effects of rising mean temperatures and increasing temperature variability (such as heatwaves) on immunocompetence of fish are not sufficiently studied to draw general conclusions. Hence, future predictions, needed for managing wild and aquaculture fish species in the face of global warming, are currently impeded. Future research should focus on the effects of realistic short-term and chronic temperature stress with a special emphasis on the most vulnerable early life stages and host–parasite interactions. Moreover, trans-generational studies are vital to elucidate the adaptation potential of fish to temperature rise (see §§4 and 5).

### (b) Hypoxia

With global warming, the dissolved oxygen (DO) levels of aquatic ecosystems are gradually declining, resulting in large-scale de-oxygenation, more frequent severe hypoxic events and drastic seasonal hypoxic declines in shallow waters (i.e. coasts, estuaries) [90–92]. Acute and chronic hypoxic conditions in aquatic environments are imposing critical challenges on the performance, behaviour, health and survival of fish [93–98]. A considerable number of studies reported that short, long-term and cyclic hypoxic conditions can compromise the fish immune system, increase the susceptibility to pathogen infections, and ultimately result in an increased risk of diseases outbreaks and mortalities (see electronic supplementary material, table S1). The immune response of fish species to short-term (2–48 h) and acute hypoxia (≤ 14 days) has been investigated in multiple studies, indicating that reduced disease resistance and survival is likely due to an impaired immunity caused by a stress response to sublethal DO levels [99–101]. For example, acute exposure to sublethal DO levels induced stress responses (e.g. increased cortisol level) in Nile tilapia (*Oreochromis niloticus*) [102,103] and channel catfish (*I. punctatus*) [104] which increased the susceptibility to bacterial infections and finally resulted in a significantly higher mortality rate. Mu *et al.* [105] reported that yellow croaker (*Larimichthys crocea*), exposed to short-term and acute hypoxia, showed downregulated transcript levels of toll-like receptors, fucolectins, and macrophage mannose receptor in the spleen and head kidney (see electronic supplementary material, table S1), suggesting a suppressed pathogen recognition. However, chronic and cycling hypoxic conditions with co-occurring steep acute hypoxic events during the summer months are more likely to represent the reality of our future oceans [90–92].

Long-term and cycling moderate hypoxia exposures (≥ 14 days) that mimic realistic scenarios were shown to cause reduced and/or delayed immune responses [94,95] and to speed up disease development [106]. Oldham and colleagues [106] reported that dial cyclic hypoxia (40–60% DO, over 22 days) accelerated the progression of amoebic gill disease and doubled cumulative mortality rates in post-smolt Atlantic salmon (*S. salar*). Chronic hypoxia was also shown to be the driving factor of either reduced or delayed immune-related gene expression towards a viral or bacterial challenge (*in vitro* and *in vivo*) in Atlantic salmon [107]. Likewise, Atlantic salmon reared under moderate hypoxia (60% DO) over 10 months and challenged with *Moritella viscosa* (agent of winter ulcer disease) after smoltification showed reduced survival with an impacted immune gene expression (e.g. lectins and acute phase proteins) in the gill and spleen of infected fish [108]. Nile tilapia reared at either low, medium, or high DO levels for 12 weeks and challenged with *Aeromonas hydrophila* showed a decreased production of oxygen radicals by leucocytes (i.e. respiratory burst) and lysozyme activity as well as lower pathogen resistance with higher mortality rates [109]. Similarly, goldfish (*Carassius auratus*) exhibit a significantly decreased activity of antioxidant enzymes and lysozymes in head kidney and spleen when subjected to chronic low DO levels, while lipid peroxidation and thus the potential for cellular damage in important immune organs increased simultaneously [110]. Finally, both acute and chronic hypoxia studies reported that fish exposed to decreased or sublethal DO levels exhibited lower specific antibody titres in circulating blood following experimental challenge with living pathogens (*Edwardsiella ictalurid, Vibrio anguillarum* or *Streptococcus agalactiae*) as compared to fish in control conditions [94,95,104,111].

In summary, the findings highlight that hypoxia compromises innate (e.g. lysozyme, phagocytic, respiratory burst activity, the abundance of C3, innate immune gene expression) and adaptive immunity (e.g. delayed antibody production, adaptive immune gene expression) resulting in a delayed or ineffective ability for pathogen clearance that is leading to a higher disease susceptibility, faster disease progression and higher mortality rates. Considering the current forecasts of climate change, hypoxia is predicted to become more widespread with more drastic seasonal declines, especially in shallow coastal waters, while pathogen abundance and virulence are increasing in warmer waters. The potential effects of hypoxia as a climate change stressor on fish immunocompetence, adaptability and migration behaviour remain severely understudied [112]. Future research should focus on the sensitivity of early life stages and the adaptive potential of different fish species to hypoxic conditions in trans-generational experiments at predicted environmental conditions (including spatial and temporal variabilities such as cycling oxygen levels) to better evaluate fish disease resistance and immunocompetence (see §§4 and 5).

### (c) Acidification

Due to climate change, the ocean sea surface pH is projected to drop on average by 0.036–0.042 (RCP2.6, if net zero was achieved by 2100) or 0.287–0.291 pH units (RCP8.5, worst case climate change scenario with a continued rise in emissions throughout the twenty-first century) by 2081–2100 compared to 2006–2015 [2]. Compared to other environmental stressors covered in this review, very few studies have investigated the effect of environmental pH on the immune response of aquatic animals.

As with the previously discussed environmental factors, in order to assess the effects of aquatic pH on an organism it is important to differentiate between acute stress and chronic stress. Furthermore, knowledge of the habitat, i.e. do the animals live in relatively stable or variable environments, is essential. In some environments, such as estuaries, fish can be exposed to drastic pH changes over a short period of time [113]. These rapid changes most probably act as a physiological stressor and would act on the immune system through the hypothalamic–pituitary–inter-renal axis [114]. Such acute pH stress (ΔpH = −0.5) was addressed in barramundi (*Lates calcarifer*) over an exposure period of 12 to 60 h and the transcriptome analysis revealed some effect on innate immunity such as lysozyme and antigen presentation [115] (see electronic supplementary material, table S1). In two studies, Bresolin de Souza and colleagues addressed the impact of chronic hypercapnia on the immune response of Atlantic halibut (*Hippoglossus hippoglossus*) [116,117]. It was apparent that 3 months of exposure to ΔpH = −0.4 resulted in downregulation of IgM heavy chain constant region, upregulation of complement component C3, activation of the alternative complement pathway, and increase in lysozyme activity in plasma. The authors suggested that the fish might be experiencing tissue inflammation due to the low pH.

In summary, it is evident that knowledge in this area of ecoimmunology is still scarce. In particular, studies addressing the interplay of immunity and pathogens are needed to evaluate infection outcomes in the hypercapnic aquatic environments. Intriguingly, CO_2_ exposure can also lead to shifts in the gut microbiota [118], which would influence the development and activity of the immune system. However, overall direct effects on the immune system seem to be limited and future studies might show that acidification is not a driving immunomodulatory factor.

### (d) Salinity

With climate change ocean surface areas in regions with net levels of evaporation will become saltier while areas with high levels of rain are expected to decrease in salinity, i.e. become fresher [119]. The changes occur both as acute and long-term changes. Acute changes will be driven by an increase in extreme weather events such as heatwaves and heavy precipitation leading to very acute and rapid changes in salinity levels. At the same time, long-term changes are caused among others by large scale changes in ocean circulation [119]. On a global scale it is forecasted that the tropical and subtropical Atlantic Ocean and the Mediterranean Sea become saltier while the Pacific and polar Arctic will decrease in salinity. For estuaries and river deltas, measurements show salinization due to saltwater intrusions and droughts [119]. Thus, when evaluating any type of physiological response to salinity changes, the life-history strategies of the species and the predicted impact on their environment need to be considered.

Most studies focus on salinity in respect to rearing protocols of euryhaline aquaculture species and less in respect to forecasted climate change induced salinity changes. Especially when investigating short and acute salinity changes, some of the observed effects are caused by physiological stress in response to the sudden change in environmental conditions rather than being a direct effect of osmolarity change on the immune system [120].

In freshwater fish, salinity levels beyond their physiological limits naturally lead to an impairment of the immune response, and to lowered survival in response to infection [121,122]. Moderate (up to 10 ppt, control 0.4 ppt, physiological maximum 15 ppt [123]) chronic (20 days) hypersalinity increased lysozyme and alternative complement pathway activity in striped catfish (*Pangasianodon hypophthalmus*) [121] (see electronic supplementary material, table S1). Nile tilapia is the most intensively studied freshwater fish species in respect to salinity effects on immunity. They can be reared in water ranging from 0–15 ppt [124]. Throughout different studies, it was shown that hypersalinity within this physiological range led to an increase in immune factors such as lysozyme activity and immune associated gene expression (*il-1b*, *cox-2*). However, other factors such as complement components were downregulated [125,126]. In general, moderate hypersalinity seemed to have activating effects on the immune system of freshwater fish, however challenge experiments with pathogens are needed to evaluate immunocompetence under this scenario.

Effects of long-term hyposalinity have mainly been studied in euryhaline fish species with optima in the brackish salinity spectrum. When salinity was reduced from 25 to 10 and 0 ppt for euryhaline *Scatophagus argus* over 4 weeks, the immune response to *A. hydrophila* infection was reduced. More specifically, cytokine gene expression was earlier and higher in 10 and 25 ppt compared to 0 ppt, and white blood cell counts were higher in high salinity [127]. In a liver transcriptome study using euryhaline (optimal 16–17 ppt, range 0–38 ppt) spotted sea bass (*Lateolabrax maculatus*) fingerlings, Zhang and colleagues [128] demonstrated that low salinity (5 ppt) led to an increase in C1q-like protein 2 (a complement protein), and interleukin 20 expression compared to high salinity (30 ppt), while other genes such as interleukin 8 and 4 were decreased (it has to be noted that in this study no control salinity was used). Interestingly, when broad-nosed pipefish (*Syngnathus typhle*) were exposed to low salinity their ability to mount an immune response to *Vibrio* bacteria was impaired [129]. All these hyposalinity studies indicate a possible resource allocation trade-off between osmoregulation and immune response as hypothesized by [129].

Salinity has an influence on the microbiota as demonstrated in Chinook salmon (*Oncorhynchus tshawytscha*). The species richness and diversity of the microbiota of fish farmed in freshwater conditions was higher than the microbiota composition in marine farmed fish [130]. However, these changes were not linked to changes in fish health. Since immune factors were not investigated, it is not possible to link microbiota composition to immunocompetence in this case.

As discussed for temperature above, it is important to consider the influence of salinity on the different life stages. We are only aware of one study investigating salinity and immunity in fish larval stages. It showed that in larvae of the anadromous species American shad (*Alosa sapidissima*) an increased salinity from 0 (control) up to 21 ppt for 60 days led to an upregulation of activity of lysozyme, superoxide dismutase and alkaline phosphatase, paired with increased survival [131].

In summary, it is difficult to identify common immune-related trends in response to salinity. While Makrinos & Bowden [34] concluded that acute salinity changes lead to an increase in phagocytosis and innate immune cells, increased susceptibility and decreased immune factors were reported in the present review. This highlights that the strength of the stressor (i.e. the difference in salinity level between control and treatment) and exposure time (gradual increase of salinity versus shock exposure) plays an important role. Hyper- as well as hyposalinity seem to have enhancing effects in some studies and inhibiting effects in other studies [34]. Generally, the observed changes seem to be species, tissue and immune factor dependent. A few studies have indicated an acclimation and adaptation potential of fish immunity to salinity [132–134] (possibly driven by genetic and epigenetic changes, see §4), hence chronic exposure studies are needed to study long-term salinity effects on disease susceptibility. Data in this area of ecoimmunology are still limited but by applying more standardized methods (see suggestions in §6) it may be possible to decipher general effects in future research.

## 3. Interactive effects of multiple climate change-related stressors

The aforementioned sections highlight effects of single climate change factors. However, it is crucial to realize that climate change is affecting multiple environmental parameters at the same time. Therefore, it is of major importance to consider and simulate scenarios that realistically reflect the future conditions and are relevant for the particular study organisms. Multi-stressor experiments allow us to understand whether environmental factors are additive, antagonistic, or synergistic and to identify which stressors are most impactful [135]. Additive effects occur when combined effects of multiple stressors equal the sum of the effects of each stressor, while antagonistic effects are less than the expected additive effect and synergistic effects have a greater combined effect than the sum of each stressor [135]. Synergism seems to be the most common outcome of multi-stressor exposure experiments indicating interactions between many stressors [135]. However, in most immune studies only changes in single environmental factors are tested which rarely reflects reality. This results in biases that are making extrapolations of lab results to the natural environment difficult. Consequently, we still have little understanding of how the simultaneous exposure to multiple (fluctuating) environmental stressors are impacting the immunity and disease resistance of fish. Below we review currently available multi-stressor experiments focussed on fish immunity and try to decipher the effect type.

Contrasting results were found in studies investigating combined effects of high temperature and acute [136] or chronic moderate hypoxia conditions [137–139] on the immune responses and disease susceptibility of fish. Chronic moderate hypoxia (approx. 70% air sat.) combined with incremental warming temperatures (12–20°C, 1°C week^− 1^) over eight weeks caused a greater impact on the hepatic innate and adaptive immune transcript expression in post-smolt Atlantic salmon (*S. salar*) as compared to high temperatures alone [138,139]. Interestingly, salmon from the same experiment kept at temperature conditions approaching their upper thermal limit (i.e. 20°C) together with chronic moderate hypoxia for additional four weeks, showed an equally high capacity to mount a robust innate immune response, when challenged with a multivalent vaccine, as compared to fish maintained at 12°C [140]. Under this climate scenario, a long-term acclimation to high temperatures and chronic moderate hypoxia did not compromise the innate immunity of Atlantic salmon [140]. It remains to be determined whether the simulated stressful conditions would have had harmful effects on the salmon's overall disease resistance if challenged with live pathogens. Remarkably, in this study persistent epigenetic changes were detected after long-term exposure to 20°C (four weeks) indicating that induced epigenetic mechanism mediated physiological stress and immune-related acclimation responses in Atlantic salmon [141] (see §4). By contrast, Wang *et al.* [137] found evidence for suppressed immunocompetence and disease resistance in vaccinated juvenile Nile tilapia (*O. niloticus*) at combined intermitted hypoxia (4.0 ± 1.0 mg l^−1^ DO) and warming (30°C versus 35°C) scenarios for two weeks. These experimental conditions led to increased higher cumulative mortality in response to *Streptococcus agalactiae* infection, both in unvaccinated and vaccinated fish. The increase in mortality was linked to a depressed immune response on the molecular level and seemed to be mainly driven by hypoxia with little impact of the additional temperature stressor. Additionally, in farmed post-smolt Atlantic salmon hypoxia (50% DO) and high temperature (16°C versus 8°C) exposure for six to seven weeks had an additive effect as alterations of immune factors were more pronounced at high temperatures [111]. These rather opposing results between the above-mentioned studies may have occurred due to various factors such as differences in (i) the species' acclimation potential, (ii) life stages, (iii) experimental designs, (iv) exposure periods of stressors, and (v) type of pathogen exposures (vaccine, viral or bacterial pathogens). This highlights the need for more investigations addressing combined stressors under naturalistic regimes appropriate for each study organism and its particular life stage (see §6).

When hypoxia is combined with salinity stress, it seems that the direction of salinity stress (i.e. hyper- versus hyposalinity) is a determining factor as seen in euryhaline *Fundulus grandis* larvae (tolerance range 0–70 ppt [50]), where high salinity (30 ppt) and hypoxia (6 mg l^−1^) had no effect on immune gene expression, low salinity (3 ppt) plus hypoxia led to a suppression of immune-related gene pathways [142]. However, as this assumption is based on only one study, we suspect that life stage, species and ecotype will have an influence on the outcome. Similarly, we are aware of only one study investigating the combination of salinity and temperature. In this study, Dawood and colleagues [122] demonstrate that hypersalinity in common carp (*Cyprinus carpio*), a freshwater fish, reduced antioxidative indices (SOD, CAT, GPx), lysozyme and respiratory burst activity. Additional acute heat stress further exaggerated this immunosuppression, indicating a synergistic effect of these two stressors. Interestingly, in the only study combining temperature and acidification, immune effects were driven by CO_2_ levels independent of exposure temperature (5, 10, 12, 14 (optimal temperature), 16, 18°C) in Atlantic halibut [143]. It therefore seems that, at least in this case, pH was the greater stressor compared to temperature [116].

In summary, with the current level of knowledge it is not possible to decipher which combination of environmental stressors affects immunocompetence in fish the most. Moreover, the species-specific tolerance level towards environmental stressors, the climate scenarios (e.g. acute, chronic, or fluctuating exposures), the age/maturity, and the sampled tissue/organ all influence the immunological outcome. Hence, it is essential to conduct future studies that realistically resemble natural multi-stressor scenarios (see §6) and to study the underlying molecular regulation mechanisms (e.g. epigenetic modifications) allowing organisms to acclimate to a changing environment. Such epigenetic changes are crucial to timely regulate physiological responses through changes in gene expression. In the next section, we review the epigenetic regulation of the immune system in response to climate change to better understand the tolerance range and immune competence of fish to cope with (newly introduced) pathogens under rapidly changing environments.

## 4. Unravelling underlying mechanisms: epigenetic regulations of immune responses

As seen in §§2 and 3, several studies indicate that fish are able to acclimate and adapt to climate change to sustain immunocompetence. Phenotypic plastic responses that enable quick acclimation to changing environments can be facilitated by epigenetic mechanisms such as DNA methylation, histone modifications (e.g. de-/acetylation, methylation), or small RNAs [144,145]. These epigenetic alterations may even be advantageous for the next generation as they can be transmitted through the germlines. Therefore, it is crucial to study these mechanisms to evaluate the acclimation potential of the fish immune system in the face of climate change.

### (a) DNA methylation

**Figure 2.**
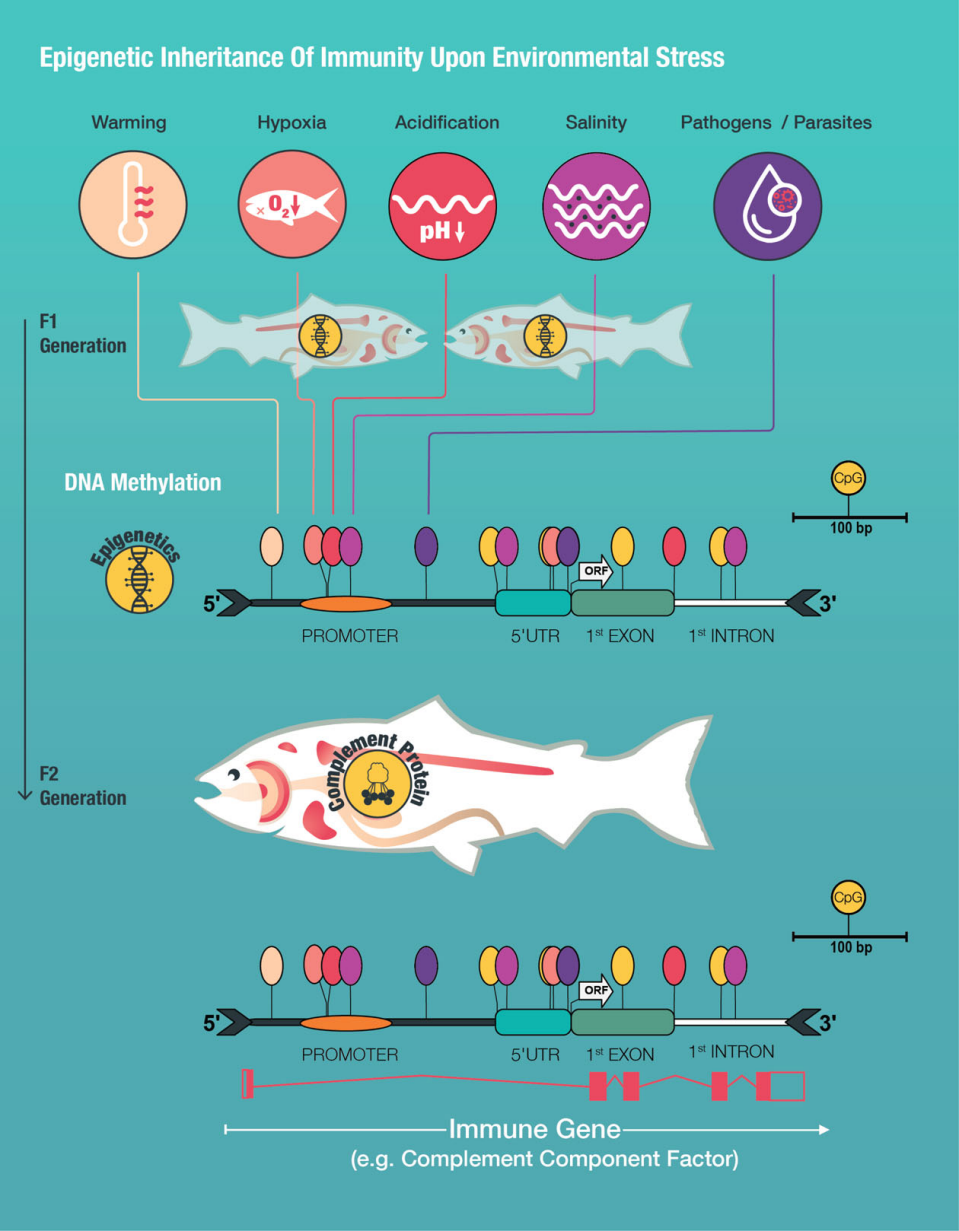
Schematic illustration of trans-generationally transmitted environmentally induced epigenetic changes (CpG methylation) around the transcription start of a hypothetical immune gene (e.g. complement component factor) modified from [141]. Depicted are CpG methylation changes (epigenetic marks) induced by environmental factors altered by climate change (warming, hypoxia, acidification, salinity, pathogens/parasites) on different genomic elements represented as a range from orange to red and to purple lollipops whereas unaffected marks are indicated as yellow lollipops. The gene structure illustration shows the putative promoter, 5′UTR, 1st coding exon, and 1st intron. The start codon and the beginning of the open reading frame (ORF) are indicated by a white arrow. The gene transcript diagram was obtained and modified from the Ensembl database showing exons as red shaded boxes and introns as lines.

DNA methylation, the reversible addition of a methyl group (CH_3_) at the 5′ carbon end of cytosines in a CpG dinucleotide context (cytosine-followed-by-guanine dinucleotide) [146], is one of the key epigenetic regulation mechanisms of gene expression, characterized by its stability and heritability across generations [147]. Various environmental factors can rapidly induce changes in DNA methylation that allow for fast adjustments in the expression of genes and thus offer an effective mechanism for immunological responses [148]. In aquatic animals, it was shown that DNA methylation is influenced by temperature [149–153], hypoxia [154–156], high temperature combined with moderate hypoxia [141], salinity [157,158], and acidification [159,160]. For example, Atlantic salmon (*S. salar*) embryos stressed with acute cold shock during larval stages, exhibit global promoter and gene-body DNA methylation changes that were associated with altered immune gene expression profiles and could have affected their immunocompetence [161]. Japanese flounder (*Paralichthys olivaceus*) exposed to acute hypoxic stimulation and re-oxygenation showed decreased DNA methylation within the promoter of two genes related to muscle immune responses that correlated with their transcript expression [156]. Further evidence was found in the studies of Beemelmanns and colleagues [138,141] with a significant downregulation of DNA methyltransferase 1 (*dnmt1*) and induced dynamic and time persistent CpG methylation marks of stress and apoptosis genes in the liver of post-smolt Atlantic salmon when exposed to hypoxia alone or combined with warming temperatures. Downregulation of *dnmt1* suggests genome-wide DNA methylation changes and a significant involvement of epigenetic regulation mechanisms in stress and immune responses of fish [138]. Persistent changes in specific CpG sites within regulatory elements (i.e. promoter, 5′UTR, 1st exon, 1st intron, figure 2) of stress and apoptosis genes after long-term heat exposure can be considered as important epigenetic marks regulating stress and immune-related acclimation responses [138]. Taken together, all these findings suggest that DNA methylation changes are important epigenetic regulation mechanisms facilitating immune responses during environmental stress exposure.

### (b) Non-coding RNAs

Transcriptional responses can be regulated through small non-coding RNAs (ncRNAs) including microRNAs (miRNAs) [162]. ncRNAs are essential to regulate temperature-driven processes [153,163–166] and miRNAs have been found to contribute to the immune response of teleost fish [167]. For example, Lau *et al*. [164] found differential levels of miRNA expression in several tissue types of marine medaka (*Oryzias melastigma*) in response to hypoxia that were linked to anti-apoptotic genes. These results indicate the key role of miRNA in regulating programmed cell death and cell survival, which is a well-known consequence of hypoxia exposure in aquatic organisms. Thus, ncRNAs are likely involved in the regulation of immune responses as indicated in mammalian studies [168].

### (c) Epigenetic memory and trans-generational immune priming

Environmentally induced epigenetic modifications (epigenetic marks) regulating immune gene expression could be an important mechanism to create physiological plasticity and hence affect immunocompetence during acclimation within a lifetime. Given that environmentally induced epigenetic marks mediating immune response are stably integrated into F_1_ germline cells and can skip epigenetic erasure/reprogramming [169], they can be transferred to the F_2_ generation. The F_2_ generation can then respond more efficiently to environmental challenges experienced previously by the F_1_ generation and could trigger local adaptation responses [152,154,160,170,171]. This process is known as ‘trans-generational (immune) priming’ (TG(I)P) [172,173]. Climate change-related environmental stressors may, for example, induce different CpG methylation patterns in immune genes of F_1_ fish that can be trans-generationally passed on to the F_2_ generation (trans-generational epigenetic priming of immune genes, figure 2) and enhance their overall immunocompetence. Epigenetic marks could therefore represent a mechanism for TGIP and facilitate the transfer of parental and even grandparental immunological experience to the next generation with the potential of mediating long-term protection [171,174,175]. Considering the current predictions for global change, this trans-generational epigenetic inheritance of immunity may allow fish to adjust their phenotype to future aquatic habitats over multiple generations [152,170,176,177]. This would provide an epigenetic time buffer for genetic adaptations (e.g. fixation of mutations within the coding region of a protein) to occur on the population level [178]. Thus, epigenetic marks can be considered as an essential regulatory mechanism for the modulation of immunological acclimation responses and may create lasting epigenetic memory [152]. Nevertheless, studies that are exploring epigenetic modifications specifically for differentially expressed immune genes in the context of climate change are so far scarce and need future attention (see §6j).

## 5. Will fish be able to adapt to maintain immunocompetence?

If and how fish species will be able to survive in future aquatic environments depends on their adaptation potential which may vary between different populations/ecotypes [38]. Three-spined sticklebacks, for example, were observed to adapt to increased temperatures within 17 generations [47]. In general, the adaptation potential depends on various factors such as the species' generation time, species’ distribution, and the pressure of other factors such as overfishing [89,179].

In zebrafish, Morgan *et al.* [180] were able to demonstrate that fish adapted to stable environments have reduced phenotypic plasticity and are less able to counter thermal effects on their metabolism and other performance indicators compared to fish from variable environments. Furthermore, the group [88] showed that evolution towards upper thermal limits is slow in zebrafish and therefore suggest a hard limit in upper thermal tolerance. Thus, fish that live close to their upper thermal limits are already struggling or will struggle to adapt to future warming (see also [89]). While these studies are not addressing immunocompetence, they highlight a limited scope in fish to adapt and thus a limit to energy resources available for immunity in changing aquatic systems.

As described in §4, phenotypic plasticity within and across generations (trans-generational plasticity or TGP) and the trans-generational parental transfer of immunity (trans-generational immune priming or TGIP) have been shown in an increasing number of fish studies and are considered as an essential mechanism to cope with rapid abiotic and biotic changes [174,175,181]. However, the adaptive potential of TGP and TGIP over many generations and their role in the adaptation process on the population level is still poorly understood. Furthermore, knowledge is lacking regarding which environmental conditions promote this phenomenon [182]. Even though it is expected that TGIP will more likely occur with adaptive benefits [175], it can also be constrained or non-specific. The latter is the case if (i) the parental and offspring environments are not matching or are unpredictable, (ii) there are mechanistic constrains of immune priming, and/or (iii) the associated energetic costs are too high (e.g. reduced fecundity and reproduction) [175,183]. Furthermore, it is still not known whether TGIP could compensate for the effects caused by multiple environmental stressors. In this context, trans-generational epigenetic marks important in regulating immune gene expression and facilitating phenotypic plasticity across many generations may be an important component triggering (local) adaptation responses on the population level. Hence, experiments simulating environmental changes in multigenerational approaches and assessing epigenetic marks in important immune genes are needed to predict the capacity of adaptive priming effects on the fish immune system.

## 6. Outlook: how can we better estimate climate change effects on fish immunity?

As highlighted throughout this review, fish immune responses and stress levels are interconnected and depend on biotic and abiotic environmental factors. Moreover, species-specific factors, life cycle stage, life history, sex, type and intensity of stressors (having possibly interacting effects), length of exposure (short, acute or chronic), and pathogens play a crucial role, making fish immune functions highly complex. Hence, analysing and comparing the alteration of fish immunocompetence upon climate change across different studies is very challenging due to a lack of comparability. For drawing a bigger picture and overall conclusions, standardized studies, taking the following aspects into consideration, are crucially needed:

### (a) Species diversity

Many authors stress that the influence of environmental factors on the fish immune response seems to be species-specific. Hence, using a set of diverse model species adapted to different environments appears to be crucial for unravelling underlying mechanisms. For example, since fish from tropical and polar regions are closer to their thermal limits than fish inhabiting temperate regions, experiments with species with broad and narrow tolerance curves (i.e. eurythermal and stenothermal) and with different generation times (different adaptation potential) are needed. To better predict climate change effects on capture fisheries and aquaculture, marine and freshwater key species from both sectors should be included.

### (b) Importance of life stage

The immune system of fish early life stages is immature making them extremely vulnerable to infection, plus they are generally more sensitive to environmental change with limited tolerance outside their optimum. While the innate immune response is functional from early on, the adaptive immune system only develops over time. Therefore, climate change effects on fish immunity will differ between life stages. Furthermore, it must be noted that the time span, until the maturation of the immune system is completed, is species-specific and temperature dependent. However, the immune response in fish larvae under climate change scenarios has scarcely been studied so far. Moreover, it is known that, for example, thermal tolerance ranges of spawners are decreased due to energy re-allocation to gamete production. It is therefore important to study different life stages as any impact on the health and disease outcome of larvae and spawners directly affects recruitment of the next generation.

### (c) Sex matters

Sexual dimorphism in fish immune defence is well described but hardly considered in climate change studies. Thus, in studies with mature fish, ideally equal ratios of females and males should be included, and the variation caused by sex should be properly accounted for in statistical models (e.g. as a covariate or random effect).

### (d) Organ diversity

Different immune organs have different functions therefore it is hard to compare immune parameters measured in different tissue samples. To get a better understanding on the (time) course of the immune response under environmental stress, it would be useful to examine the most important immune organs (head kidney, thymus, spleen, lymphoid-associated tissues) and blood.

### (e) Baselines and comparability

We found that in many publications the experiments are poorly described with crucial information on the species and/or experimental conditions is missing. For instance, the species' physiological optimum temperature (which may vary between seasons) needs to be reported and should be applied as a control in experimental designs. Furthermore, the animal's habitat (e.g. variable or stable environments), in regard to the factors tested, needs to be considered. The use descriptions such as ‘lower/decreased’ or ‘higher/increased’ experimental temperatures/DO/salinity/pH should be avoided without reporting the species' natural preferences and range. Limited information can be misleading, especially when the temperature of the control group does not match the temperature of the species’ physiological optimum or the natural habitat. Moreover, analytical methods should be standardized, and attention needs to be paid to technical details such as performing measurements (e.g. ELISA) at the experimental temperature.

### (f) Experimental replication

To analyse experimental data, suitable statistical models must be applied which require an appropriate number of replicates. During this literature review it was noted that in many studies treatments are not sufficiently replicated. Often treatments were not replicated or only duplicated (i.e. per treatment group only one or two tank replicates were used). Consequently, statistical analyses are not performed based on replicates but on pseudoreplicates (fish sampled from the same tank) as if they were real replicates, leading to invalid results. When working with small numbers of tank replicates, pseudoreplication (fish sampled from the same tank) may be necessary but has to be statistically accounted for by using e.g. generalized mixed effect models which include not only fixed factors (the stressor being examined such as temperature, hypoxia, etc.) but also ‘tank’ as a random factor (to account for potential tank effects). Especially in larval experiments, potential treatment effects are easily masked by the high individual variability and difficult to be statistically detected. Hence, experiments should be designed in a manner that maximizes statistical power by increasing the number of tank replicates (note: the minimum for physiological studies are three tank replicates but for molecular studies more replicates are needed). See also [184] for guidance.

### (g) Long-term studies

Acute responses to extreme weather events can be experimentally measured over short periods of time. However, to assess potential mid- and long-term effects of these events, fish need to be monitored over longer periods of time (days to weeks) after the end of the heat/cold stress. To examine chronic climate change effects as well as the acclimation and adaptation potential of different fish species, more long-term (if possible trans-generational) experiments are needed.

### (h) Multi-stressor experiments

To date we still have little understanding of how the simultaneous exposure to multiple stressors at fluctuating regimes is impacting fish immunity and disease resistances. Such knowledge is crucial, as the severity and duration of several critical environmental stressors (e.g. warming, hypoxia, acidification, and/or others) are predicted to increase in the aquatic environment due to ongoing global climate change. In future experimental research studies, it is thus of major importance to simulate multi-stressor scenarios that realistically reflect present and/or future conditions of the study organism. Since the fish immune response appears to be pathogen dependent, ecologically and economically relevant pathogens should be included in studies to examine disease resistance and survival under climate change scenarios. Additionally, the influence of environmental pollution should be experimentally evaluated. In general, toxicity of pollutants (pesticides, heavy metals, persistent organic pollutants) in aquatic environments is increased through warming and acidification [185]. However, these studies rarely consider the immune response of the animals. Moreover, experimental biases (e.g. fish size, age, maturity, sex, density, tank size, shape etc.) need to be considered and minimized. Studies that resemble natural multi-stressor scenarios as realistically as possible would allow us to draw more meaningful conclusions regarding the disease resistance of fish in the future.

### (i) Integration of methods, meta-analyses and predictive models

Integrative studies that combine standard immune assays, transcriptomics, proteomics, epigenetics and microbiome analyses are crucially needed to move beyond measuring only fragments of the immune response and to better elucidate the relation between immune and stress response. The overall goal is to identify biomarkers that allow us to better predict the species' acclimation and adaptation potential to future conditions and hence their disease resistance (see also §6j).

Unfortunately, measuring the expression of individual genes does not necessarily contribute to our understanding of the overall immune response particularly when the target genes are not key genes. For a more complete picture, transcriptomics is a suitable tool, especially when studying early life stages to unravel the ontogeny of the immune system in response to environmental factors. However, the expression of a gene does not necessarily lead to protein synthesis due to post-transcriptional regulation of mRNA resulting e.g. in a downregulation of the protein expression [167]. By contrast, the proteome is a close approximation of the organism's response to environmental stressors [186]. Furthermore, the microbiome is influenced by changing environmental conditions while being interrelated with the immune system. Hence, microbiome analyses may be useful to elucidate underlying mechanisms of climate change effects on fish immunity.

Moreover, statistical meta-analyses of climate change effects on fish immunity are crucially needed for drawing general conclusions and providing an overarching picture. Meta-analyses may be used to evaluate the effect of single or multiple, acute or chronic climate change effects on either the innate, adaptive or overall fish immune response. Data synthesis would further allow us to determine, for example, if certain species, genera or ecotypes differ from other groups and to identify predictors that can explain these differences. Ultimately, the development of predictive next-generation climate-disease models is essential to project the disease resistance and adaptation potential of fish to future environmental conditions [187].

### (j) Development of novel epigenetic immune biomarkers to predict future disease outcomes

Epigenetic marks such as CpG methylation changes are highly sensitive to environmental stressors and if they are stably associated with phenotypic traits (e.g. immune responses, disease resistance), they can serve as epigenetic biomarkers (EBs). In human medicine, EBs are already applied as non-invasive diagnostic tools for the prognosis of cancer and other diseases [188]. The new development of non-invasive epigenetic biomarkers to predict future phenotypic traits (e.g. disease resistance) in aquatic organisms in the context of climate change and aquaculture has recently drawn increasing attention. In principle, EBs allow for the identification of (i) early pathogen exposures, (ii) start of detrimental infections, and (iii) predictions of disease outcome and survival. The implementation of epigenetic health markers in fish would contribute critically to a time-efficient disease assessment and could help to reduce the risk of disease outbreaks, parasite infections and/or mass mortality events in aquacultures. Consequently, the establishment of EBs for fish health would prove extremely valuable as diagnostic, predictive and prognostic tools. EB tools can help to improve mitigating strategies and risk management caused by climate change-related immune challenges. Ultimately, EBs may support biomonitoring, ecological conservation programmes, and a more sustainable aquaculture management in the future [189,190].

## 7. Conclusion

**Figure 3.**
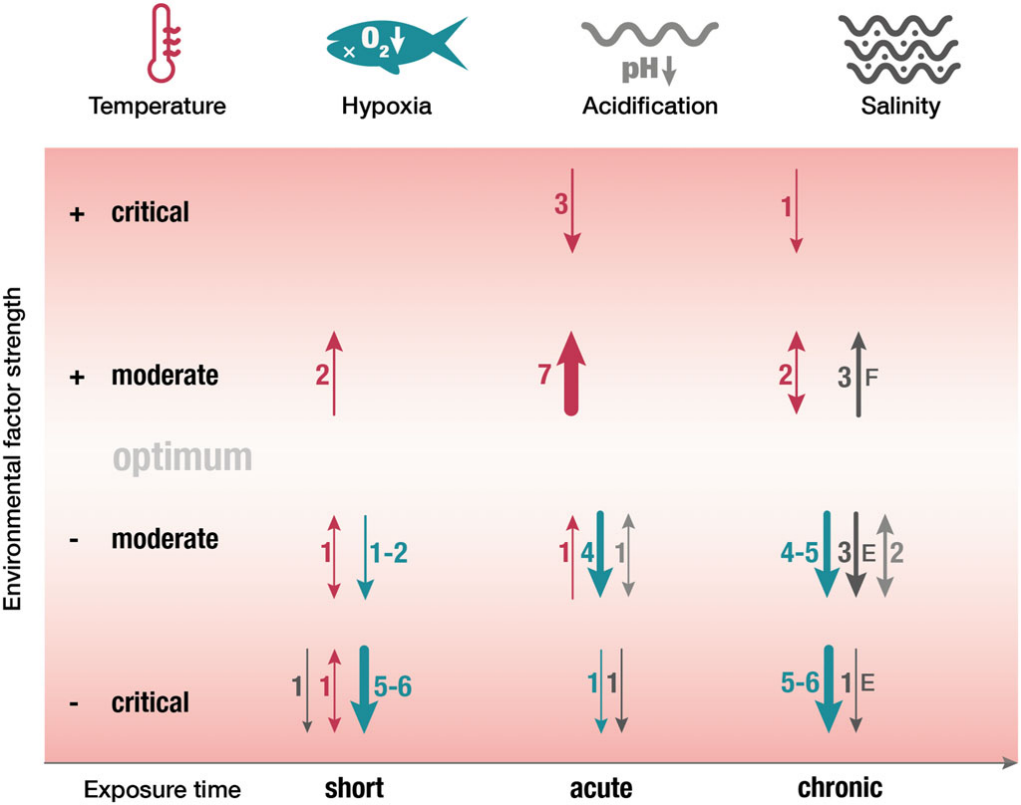
Schematic illustration of estimated climate change effects on fish immunity highlighting the direction and reliability of observed effects. Environmental factors are temperature = red, hypoxia = green, acidification = light grey, salinity = dark grey. Factor strength is generalized as moderate and critical. Exposure time is summarized as short (less than 48 h), acute (48 h to two weeks) and chronic (more than two weeks). Upward arrows represent an overall increase in immunity; downward arrows indicate a general decrease in immunity (both measured as disease survival, enhanced/decreased immune factors). + and – indicate the direction of diversion from the optimum. For salinity studies: E = euryhaline, F = freshwater fish. Arrow thickness and numbers represent the number of included publications (cited in this review, shown in electronic supplementary material, table S1, as well as [34] and [38]).

To answer the question if fish will be immunocompetent enough to fight infections in the face of climate change, we here reviewed studies investigating the effect of changing environmental factors in single and multi-stressor experiments assessing the fish immune response and disease resistance (see electronic supplementary material, table S1). Temperatures outside the thermal optimum and hypoxia can suppress the immune response either directly through the sensitivity of immune factors or indirectly through an increased stress response and/or disadvantageous alterations of the microbiome. Consequently, acute and chronic temperature and oxygen shifts may lead to immunological disorders and an increased susceptibility to infections in fish (figure 3). Considering that rising temperatures have also been shown to increase the infectivity and virulence of pathogens, this could be detrimental to fish health and therefore lead to higher mortality rates. For acidification and changes in salinity, comprehensive conclusions regarding their effect on fish immunity can currently not be drawn since acidification studies are scarce and salinity studies show contrasting results (figure 3). However, climate change does not affect only single environmental factors as warming, hypoxia, acidification and salinity changes can occur simultaneously. Hence, multi-stressor experiments simulating future scenarios are essential since it is not possible to decipher the effect of various stressors on the fish immunocompetence with the current level of knowledge. Nonetheless, since single stressors such as changing temperature or hypoxia alone have already been demonstrated to have adverse effects on fish immunity and disease resistance, the interacting effects of multiple stressors can be expected to be even more harmful.

To better understand the acclimation potential of fish coping with pathogens in rapidly changing environments, underlying molecular regulation mechanisms have to be unravelled. Epigenetic changes can be considered as an essential regulatory mechanism for the modulation of immunological responses in fish. Lasting epigenetic memory may even provide a time buffer for genetic adaptations to occur on the population level. Yet, studies that are exploring epigenetic modifications of fish immunity in the context of climate change are so far scarce and need future attention.

In general, it can be concluded that the impact of changing environments on fish health will depend on the fish species, their tolerance strategy, the ecotype, the life cycle stage, the infecting pathogen/parasite and the specific climate scenario (e.g. acute, chronic, or fluctuating exposure). Fish that are adapted to very stable environments and live close to their physiological maximum (e.g. in the tropics) might have a limited immunocompetence as they will have to reallocate energy to maintain homeostasis.

In conclusion, future predictions regarding fish immune functions are currently difficult to draw mainly due to the lack of multi-stressor studies. Hence, it is essential to conduct studies that realistically resemble future scenarios including all key stressors and pathogens/parasites with a special focus on the sensitivity of early life stages. Additionally, trans-generational experiments to unravel the underlying molecular mechanisms allowing fish to acclimate and potentially adapt to changing environments are key for the prediction of the immunocompetence and diseases resistance of fish in the future. Ultimately, the development of predictive climate-disease models and epigenetic biomarkers is essential to project if fish are immunocompetent enough to face climate change.

## Data Availability

Electronic supplementary material, table S1 is provided in [191].
